# An Evaluation of Social Bridging and Bonding Mechanisms in the Association of Social Networks and Cognitive Function

**DOI:** 10.1093/geroni/igab046.2194

**Published:** 2021-12-17

**Authors:** Siyun Peng, Brea Perry, Adam Roth, Max Coleman, Hope Sheean

**Affiliations:** 1 Indiana University, Bloomington, Indiana, United States; 2 Indiana University, Indianapolis, Indiana, United States

## Abstract

Background and Objectives: Substantial evidence links social connectedness prospectively to cognitive aging outcomes, but there is little agreement about the social processes or mechanisms that drive this relationship. This study evaluated nine measures of social connectedness, focusing on two distinct forms of social enrichment – access to an expansive and diverse set of loosely connected individuals (i.e., social bridging) and integration in a supportive network of close ties (i.e., social bonding). Research Design and Methods: This study used egocentric social network and clinical cognitive data from 311 older adults in the first wave of the Social Networks in Alzheimer Disease (SNAD) study. Linear regressions adjusting for gender, age, education, and depression symptoms were used to estimate the association between nine measures of social connectedness and global cognitive function, verbal memory, and attention. Results: Measures indicative of social bridging (larger network size, lower density, presence of weak ties, and proportion non-kin) were consistently associated with better cognitive outcomes, while measures of social bonding largely produced null effects. Discussion and Implications: These findings suggest that the protective benefits of social connectedness for cognitive function and memory may operate primarily through a cognitive reserve mechanism that is driven by irregular contact with a larger and more diverse group of peripheral others. Population-level interventions that promote the cultivation of social bridging relationships and activities may have benefits for cognition later in life.

